# Clinical-Pathological Conference Series from the Medical University of Graz: Case No 171: A 37-year-old engineer with bolus hold-up (esophageal food impaction)

**DOI:** 10.1007/s00508-020-01694-x

**Published:** 2020-06-29

**Authors:** Elisabeth Fabian, Hans Peter Gröchenig, Philipp K. Bauer, Andreas J. Eherer, Markus Gugatschka, Lukas Binder, Cord Langner, Peter Fickert, Guenter J. Krejs

**Affiliations:** 1 Division of Gastroenterology and Hepatology, Department of Internal Medicine III, Medical University of Vienna, Vienna, Austria; 2 Department of Internal Medicine, Hospital Brothers of St. John of God, Sankt Veit an der Glan, Austria; 3 Division of Infectious Diseases and Tropical Medicine, Department of Internal Medicine I, Medical University of Vienna, Vienna, Austria; 4 Division of Gastroenterology and Hepatology, Department of Internal Medicine, Medical University of Graz, Auenbruggerplatz 15, 8036 Graz, Austria; 5 Division of Phoniatrics, Department of Otorhinolaryngology, Medical University of Graz, Graz, Austria; 6 Department of Pathology, Medical University of Graz, Graz, Austria

**Keywords:** Dysphagia, Eosinophilic esophagitis, Budesonide, Proton pump inhibitors

## Presentation of case

### Dr. L. Binder:

The patient is a technical engineer working for a large international company. Except for recurrent episodes of dysphagia over the last 3 years, his history is unremarkable. He reports that “ingested food gets stuck behind his chest bone about once a month”. Originally from Brno, Czech Republic, he has been living in France for the past several years where he has been treated for dysphagia by otorhinolaryngologists who prescribed prokinetics, a proton pump inhibitor (PPI) and neuroleptics; however, these medications did not improve his dysphagia. Finishing a meal would take him about twice as long as his wife. After moving to Graz, Austria 3 months ago, he again experienced a bolus hold-up (a piece of meat got stuck in his esophagus). Used to being treated by otorhinolaryngologists, the patient came to the emergency room of the Department for Otorhinolaryngology of this institution where the bolus was removed with the patient under general anesthesia using a rigid esophagoscope. The investigation also showed a questionable stenosis of the proximal esophagus with marked vulnerability of the mucosa. The patient was told that surgery might become necessary if esophageal injury or perforation has occurred, and he was admitted for observation. The physical examination was otherwise unremarkable and all routine laboratory parameters were within normal limits.

A diagnostic test was performed.

## Differential diagnosis

### Dr. H.P. Gröchenig:

The patient under discussion is a young man with a history of dysphagia and recurrent food impaction, which may be due to a stenosis of the esophagus with remarkably increased vulnerability of the mucosa seen on endoscopy. Prokinetics, PPIs and neuroleptic drugs did not improve his condition. Dysphagia is defined as a sensation of sticking or obstruction of the passage of food through the mouth, pharynx or esophagus and should be distinguished from other symptoms, such as aphagia and odynophagia. Aphagia is the complete esophageal obstruction, which is usually due to bolus impaction and represents a medical emergency. Odynophagia means pain on swallowing and frequently occurs together with dysphagia [1]. The prevalence of any type of dysphagia in the general population is very high and amounts to about 20%. It affects more often women and older persons than men and younger people [2]. The differential diagnosis in patients with dysphagia is broad and involves a variety of underlying mechanisms and conditions (Table 1). Dysphagia can be categorized by location, i.e. oropharyngeal (or transfer) dysphagia and esophageal dysphagia, and by cause. Determination of whether the problem is associated with difficulty transporting oral contents through the pharynx into the esophagus or within the esophagus is crucial for the diagnostic evaluation. Dysphagia caused by a large bolus or luminal narrowing is called mechanical dysphagia, whereas dysphagia due to weakness of peristaltic contractions or due to impaired deglutitive inhibition causing nonperistaltic contractions and impaired sphincter relaxation is termed motor dysphagia [1]. A detailed history and an awareness of potential diagnoses are useful in identifying true esophageal dysphagia. Indeed, oropharyngeal dysphagia can be distinguished from esophageal dysphagia with 80% accuracy if there is (1) delay in initiating the swallow, (2) deglutitive postnasal regurgitation, (3) deglutitive coughing and (4) repetitive swallowing needed to achieve satisfactory clearance [3, 4]. Since these symptoms are not reported in the discussed patient, oropharyngeal dysphagia can most likely be ruled out as the cause of his problem.

**Table 1:** Differential diagnoses of dysphagia [1]

	Oropharyngeal dysphagia		Esophageal dysphagia	
Propulsive	*Neurogenic* Cerebral vascular accident Parkinson’s disease Amyotrophic lateral sclerosis Brainstem tumor Guillain-Barré syndrome Huntington’s chorea Post-polio syndrome Multiple sclerosis Cerebral palsy	*Myogenic* Myasthenia gravis Polymyositis Mixed connective tissue disease Oculopharyngeal muscular dystrophy Paraneoplastic syndrome Myotonic dystrophy Sarcoidosis	Dysphagia for solids and liquids	
			GERD with weak peristalsis Achalasia (primary and secondary) Diffuse esophageal spasm Scleroderma	
Structural	Zenker’s diverticulum Neoplasm Cervical web Cricopharyngeal bar Osteophytes Congenital abnormalities Post head and neck surgery Chemotherapy mucositis Radiation Corrosive injury Infection		Dysphagia for solids	
			*Intermittent* Schatzki ring Esophageal web *Progressive* Neoplasm *Variable* Peptic stricture Eosinophilic esophagitis Hiatal hernia Extrinsic compression Surgical stenosis Radiation esophagitis Ringed esophagus Congenital esophageal stenosis	*Odynophagia* Pill esophagitis Infectious esophagitis Caustic injury Chemotherapy Sclerotherapy Crohn’s disease Behçet’s disease Bullous pemphigoid Lichen planus

*GERD* gastroesophageal reflux disease

For identification of potential causes of esophageal dysphagia, a focused history should be taken. In particular it is important to distinguish whether (1) dysphagia is experienced when swallowing solids and liquids or with solids alone, (2) it is progressive or (3) there are other associated symptoms such as pain on swallowing or regurgitation [2]. This will help identify an obstructive or functional cause of dysphagia. Motor disorders such as achalasia and esophagogastric junction outflow obstruction, and major disorders of peristalsis such as absent contractility, distal esophageal spasm and hypercontractile esophagus (jackhammer) are typically associated with bolus hold-up causing dysphagia. Minor motility disorders such as ineffective esophageal motility and fragmented peristalsis are, however, of uncertain importance in dysphagia [5, 6]. The discussed patient reported intermittent bolus hold-up events when swallowing solid food without progression of symptoms or weight loss over time; swallowing liquids does not cause problems. The following three essential diagnostic work-up modalities are available: (1) esophagoscopy with esophageal biopsies to exclude fibrous strictures, reflux esophagitis and eosinophilic esophagitis, (2) barium swallow, preferably as cineradiography, to exclude subtle strictures missed on endoscopy and to observe bolus progression, and (3) esophageal manometry to detect motor disorders. In the case of negative endoscopy and unremarkable manometry, the diagnosis of functional dysphagia, which is caused by factors such as medication (opioids and other motility-altering drugs [7]), gastroesophageal reflux, sensitization of the peripheral nerves or central processing abnormalities (including psychological factors) has to be considered [2]. In view of the patient’s history, some specific differential diagnoses should be discussed:

With a prevalence of 10–20% in the western world, gastroesophageal reflux disease (GERD) is the most common gastrointestinal diagnosis reported during visits to outpatient clinics [8]. Clinically, heartburn and regurgitation are the leading symptoms in GERD, but also extraesophageal manifestations such as chronic cough, asthma, laryngitis and other airway symptoms as well as atypical symptoms such as dyspepsia, epigastric pain, nausea, bloating and belching may be present (Table 2). With respect to the esophagus, the spectrum of injury includes esophagitis, stricture, the development of columnar metaplasia (Barrett’s esophagus) and adenocarcinoma [9]. GERD is more frequently linked to dysphagia than any other cause [10]. Although dysphagia can be associated with uncomplicated GERD, its presence warrants investigation for potential complications such as underlying motility disorder, peptic stricture, narrow Schatzki ring, or malignancy [11]. In patients with excessive reflux of acid and pepsin, injury of the surface layers of esophageal mucosa and consequently erosive esophagitis will occur [9]. The severity of esophagitis has been shown to be even more important than the actual peptic stricture diameter in generating symptoms of dysphagia [12]. The diagnosis of GERD is made based on a particular constellation of symptoms, endoscopic findings, pH-metry, and response to acid-suppressive therapy. Barium radiographs, routine biopsies and esophageal manometry have no role in the diagnosis of GERD [13].

**Table 2:** Symptoms associated with gastroesophageal reflux disease (GERD) [9]

Esophageal symptoms	Extraesophageal symptoms
Common symptoms: Heartburn Regurgitation Dysphagia Chest pain	Chronic cough
	Laryngitis (hoarseness, throat clearing)
	Asthma (reflux as a cofactor leading to poorly controlled disease)
	Erosion of dental enamel
	Proposed associations: Pharyngitis Sinusitis Recurrent otitis media Idiopathic pulmonary fibrosis
Less common symptoms: Odynophagia Water brash Subxiphoidal pain Nausea	

In clinical practice, dysphagia is also frequently associated with the administration of certain drugs including doxycycline, tetracycline, bisphosphonates, iron preparations and nonsteroidal anti-inflammatory drugs, which can directly damage the esophageal mucosa. This condition is termed pill esophagitis. It is often accompanied by odynophagia, i.e. the sensation of retrosternal pain as the bolus passes [14, 15] and is found in younger patients. However, pill esophagitis typically does not induce intermittent symptoms and is usually not responsible for food impaction as reported in the discussed patient.

Indeed, the most common diagnosis in young patients who present with dysphagia for predominantly solid food, recurrent food impaction and chest pain is eosinophilic esophagitis. It is a chronic and abnormal Th2-type immune response characterized by intense eosinophilic inflammation in the esophageal epithelium, leading to esophageal dysfunction, remodeling of the esophageal wall accompanied by subepithelial fibrosis [16], and subsequent esophageal dysmotility [17]. The prevalence of this disease has been increasing over the past decades and is estimated to be 23 per 100,000 in North American and European populations [18]. Eosinophilic esophagitis has been reported in all ages, but is predominantly found in young men aged 20–40 years and in children [19] who often present with a history of atopy including asthma, allergic rhinitis, atopic dermatitis and immunoglobulin (Ig) E‑mediated food allergy [20]. The most common symptoms in the affected adults are dysphagia, reported by 25–100% of patients, and food impaction, found in 33–50% of patients [21]. The diagnosis of eosinophilic esophagitis is often delayed since about 30% of patients may experience some benefit from PPIs. Eosinophilic esophagitis must be differentiated from secondary eosinophilia and eosinophilic gastroenteritis involving the entire gastrointestinal tract [16]. On endoscopy, linear furrows, concentric rings (trachealization, ringed esophagus, cat esophagus), white exudates, decreased capillary vasculature in the esophageal mucosa, esophageal strictures and a narrow-caliber esophagus are characteristic, but not specific findings in eosinophilic esophagitis [22]. For histological proof of eosinophilic esophagitis, more than 15 eosinophils per high-power field (HPF) in the epithelium are required [23]. The management of eosinophilic esophagitis consists of swallowed topical corticosteroids, particularly budesonide. Empirical elimination diets are another effective therapeutic approach and should be tried. Esophageal dilatation of strictures that persist after drug therapies and diet may be necessary [20].

Another differential diagnosis that should be considered in a young patient with dysphagia is achalasia. Achalasia is a rare primary motility disorder of the esophagus caused by the loss of nitric oxide and vasoactive intestinal polypeptide releasing inhibitory interneurons in the myenteric plexus that are involved in facilitating lower esophageal sphincter (LES) relaxation for gastric accommodation of food boluses [24–26]. This results in aperistalsis in the tubular esophagus and impaired relaxation of the LES [27]. The pathogenesis of achalasia is not yet fully understood. Loss of the inhibitory innervation of the esophagus can be due to extrinsic (including central nervous lesions involving the dorsal motor nucleus or the vagal fibres) or intrinsic causes, i.e. loss of the inhibitory ganglion cells in the myenteric plexus due to inflammation with subsequent progressive destruction of the myenteric ganglion cells and neural fibrosis [28]. Degeneration of inhibitory postganglionic neurons of the esophagus and LES due to inflammation [29, 30] may eventually lead to impaired relaxation of the LES and hypercontractility of the distal esophagus [31]. Typical symptoms of achalasia include dysphagia for solids and liquids in up to 100% [32–34], regurgitation of undigested food, chest pain [35], weight loss and nocturnal cough [32] as well as belching due to alterations of the upper esophageal belch reflex [36], and hiccups [37]. Dyspepsia and the sensation of heartburn which is reported by 72% of affected patients [38] often lead to a misdiagnosis of GERD [39]. For the diagnosis of achalasia, barium esophagogram and endoscopy are mandatory in addition to manometry. By definition, the manometric finding of aperistalsis and incomplete LES relaxation without evidence of mechanical obstruction solidifies the diagnosis of achalasia [40]. The incidence of achalasia is 1 in 100,000 individuals per year with a peak between 30 and 60 years of age; the prevalence is about 10 per 100,000 population [41–43]. In view of all of the above, I do not think that this patient’s presentation speaks for the diagnosis of achalasia. In rare cases, achalasia may be mimicked by cancer (pseudoachalasia), which we have recently described in a very young patient [44]. However, the long history of dysphagia without symptom progression speaks against this diagnosis.

Rarely, dysphagia found in younger patients may be caused by congenital abnormalities of the aortic arch such as double aortic arch, right or cervical aortic arch, Kommerell’s diverticulum, an aberrant right subclavian artery (arteria lusoria) or pulmonary artery sling. On barium swallow, the bayonet sign is typical for dysphagia aortica or dysphagia lusoria. In the case of suspected abnormal vascular anatomy, CT angiography will confirm the diagnosis [45].

However, the particular constellation of factors in this patient, namely the history, his age, the intermittent episodes of dysphagia and food impaction, and the fact that he did not respond to PPI therapy strongly suggests the diagnosis of eosinophilic esophagitis. Thus, esophageal biopsies would help establish an unequivocal diagnosis.

## Dr. H.P. Gröchenig’s diagnosis

- Eosinophilic esophagitis

## Discussion of case

### Dr. M. Gugatschka:

**Fig. 1:**
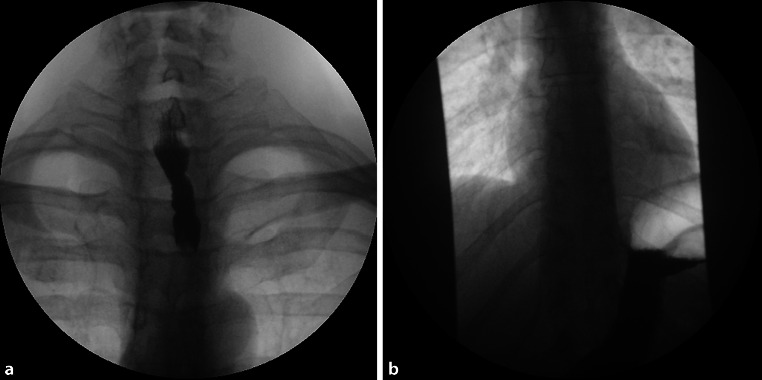
Images from a barium swallow (cineesophagogram) with **a** complete hold-up of barium in the proximal esophagus and **b** free passage of barium into the stomach after removal of the bolus, without evidence of perforation

When the discussed patient came to the emergency room, the diagnosis of food impaction was confirmed by esophagogram (Fig. 1). After the bolus had been removed by means of a rigid esophagoscope, the patient had a normal barium swallow without signs of achalasia.

### Dr. A.J. Eherer:

**Fig. 2:**
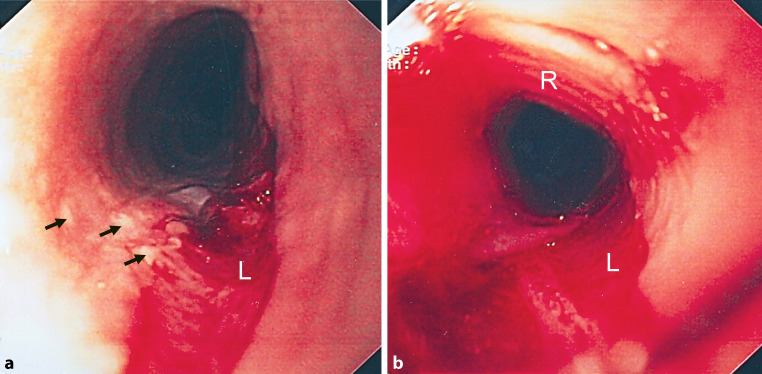
Esophagoscopy shows white plaques (*arrows*) and a laceration (L) with sloughed-off mucosa and some bleeding. Discrete circular rings (R) are also visible

Manometry revealed a slightly prolonged duration of swallowing with minimal nonspecific alterations, probably due to mild esophageal spasm. On endoscopy, multiple areas of the esophagus appeared like crepe paper with thickened mucosa, there were white plaques, longitudinal furrows and minimal circular rings (Fig. 2). Biopsies were taken from multiple sites in the distal 10 cm of esophagus.

### Dr. C. Langner:

**Fig. 3:**
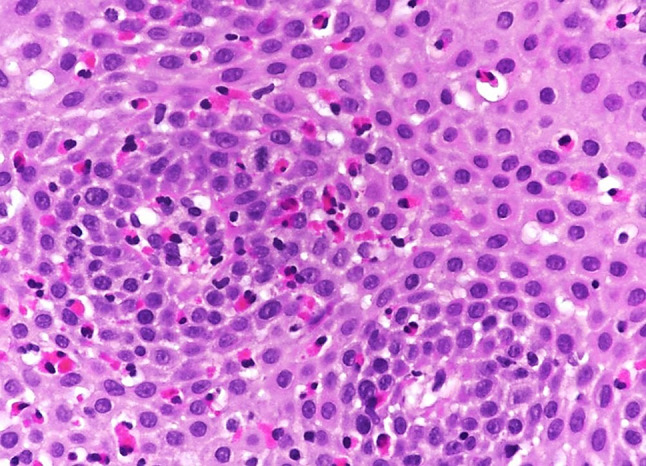
The esophageal biopsy shows an increased number of eosinophils (>15 per high-power field, HPF) between squamous epithelial cells. There is also basal zone hyperplasia and dilatation of intercellular spaces, i.e. spongiosis (original × 200, hematoxylin & eosin stain)

Histology of the esophageal biopsies revealed eosinophilic esophagitis (Fig. 3), which is a chronic, clinically and histologically defined, inflammatory condition of the esophagus that should be differentiated from other diseases that cause or potentially contribute to esophageal eosinophilia (Table 3), such as GERD, hypereosinophilic syndrome, Crohn’s disease, celiac disease, connective tissue disease, achalasia, drugs and infection. Of these, GERD is considered to be the most common cause of secondary esophageal eosinophilia in clinical practice [47–50]. The histological hallmark of eosinophilic esophagitis is a marked, often patchy infiltration of the esophageal epithelium with more than 15 eosinophils per HPF. Qualitatively, the eosinophilic distribution is diffuse throughout the epithelium without superficial or basal predominance. Furthermore, eosinophil degranulation, aggregates of 5–10 eosinophils, eosinophilic surface layering with microabscesses (visible as white plaques on endoscopy), basal zone hyperplasia, papillary elongation, dilated intercellular spaces, and increased eosinophils in stroma with fibrosis of the lamina propria may be present [23, 51, 52]. Due to the marked variability in the eosinophil counts in both per biopsy and per HPF counts, multiple biopsies from different locations need to be obtained to confirm the diagnosis [52]. Characteristic endoscopic findings such as furrows and exudates are associated with a higher peak eosinophil count and should therefore be targeted; macroscopically normal mucosa shows the lowest eosinophil counts [53]. For this reason, current guidelines recommend that at least six biopsies be taken from different locations, focusing on areas with endoscopic mucosal abnormalities [23].

**Table 3:** Conditions associated with esophageal eosinophilia [46]

Eosinophilic esophagitis
Eosinophilic gastritis, gastroenteritis, or colitis with esophageal involvement
Gastroesophageal reflux disease
Achalasia and other disorders of esophageal dysmotility
Hypereosinophilic syndrome
Crohn’s disease with esophageal involvement
Infections (fungal, viral)
Connective tissue disorders
Hypermotility syndromes
Autoimmune disorders and vasculitides
Dermatologic conditions with esophageal involvement (i.e. pemphigus)
Drug hypersensitivity reactions
Pill esophagitis
Graft vs. host disease
Mendelian disorders (Marfan syndrome type II, hyper-IgE syndrome, *PTEN*-hamartoma tumor syndrome, Netherton’s syndrome, severe atopy metabolic wasting syndrome)

*PTEN*

### Dr. L. Binder:

The human esophagus is lined by a stratified squamous epithelium that is devoid of eosinophils under normal conditions [52]. The pathogenesis of eosinophilic esophagitis is not completely understood, but it is suggested that an enhanced Th2-type immune reaction against causal food allergens, with or without a potential trigger by aeroallergens, is the main underlying mechanism [54, 55]. This reaction occurs primarily via non-IgE-mediated hypersensitivity. When esophageal epithelium is exposed to causal allergens, they will permeate the subepithelium and trigger the activation of dendritic cells through the induction of thymic stromal lymphopoietin (TSLP) [56]. Activated dendritic cells then strongly induce the proliferation of Th2 cells, which leads to an increase in cytokines associated with eosinophilic inflammation [54]. These include interleukin (IL)-5, which differentiates and recruits intramedullary eosinophils or those in the peripheral intravascular compartment [57], and IL-13 and IL-15, which induce secretion of eotaxin‑3 from epithelial cells, one of the strongest chemotactic factors for eosinophils [58]. In addition, IL-13 decreases the barrier function of the squamous epithelium by decreasing gene expression of the epidermal differentiation complex [59]. In conjunction with mast cells, aggregated and activated eosinophils produce transforming growth factor(TGF)-β1. Together with activated fibroblasts and periostin this subsequently leads to fibrotic changes in the esophageal wall and dysfunction of the smooth muscles. Due to the finding of single nucleotide polymorphisms in genes encoding eotaxin‑3, TGF-β1, filaggrin, TSLP and TSLP receptor, a genetic susceptibility to eosinophilic esophagitis is suggested [54].

Clinical symptoms of eosinophilic esophagitis differ between children and adults. Children primarily present with nonspecific symptoms such as heartburn, nausea, vomiting, abdominal pain or failure to thrive in addition to dysphagia. Since the disease progresses over time with subsequent fibrostenotic changes of the esophageal wall, adults mostly complain of eating difficulties, repeated dysphagia and food impaction [60]. Prolonged mastication, ample use of liquids to wash down solids, and prolonged time to finish a meal are consequences frequently found in patients with eosinophilic esophagitis [20]. The acronym IMPACT summarizes adaptive behaviors related to longstanding dysphagia: Imbibe fluid with meals, Modify food (cutting into small pieces, pureeing), Prolong meal times, Avoid hard texture foods, Chew excessively, Turn away tablets/pills [61]. As already mentioned by Dr. Gröchenig, linear furrows, concentric rings (trachealization, ringed esophagus, cat esophagus), white exudates, decreased vasculature in the esophageal mucosa, esophageal strictures, a narrow-caliber esophagus, increased mucosal vulnerability and mucosal edema are characteristic, but not specific findings in eosinophilic esophagitis [22].

Management of eosinophilic esophagitis includes empiric elimination diet, pharmacotherapy and endoscopic dilatation of strictures. Different types of diet therapy including elemental diet, allergy testing-based diet and empiric elimination diet have been tested in patients with eosinophilic esophagitis. Since several approaches have turned out to lack compliance for long-term use, and data reported that the skin prick test predicted only 13% of foods associated with eosinophilic esophagitis, an empiric elimination diet in which the most common allergens are excluded is currently recommended for adults as a more practical method [16, 62]. A recent meta-analysis found histological remission in 70% of patients on the six-food group elimination diet (wheat, milk, eggs, nuts, soy, seafood) and in 50% of patients on the four-food group elimination diet (dairy, eggs, legumes, wheat) [63]. Thus, empiric elimination diet may be a useful therapeutic approach and may allow reduction or discontinuation of medications in patients with eosinophilic esophagitis.

A significant number of patients with eosinophilic esophagitis are on treatment with PPIs with at least 30% exhibiting both symptomatic and histological improvement [64]. This is because eosinophilic inflammation may also occur as a consequence of GERD suggesting another disease entity than eosinophilic esophagitis called proton pump inhibitor-responsive esophageal eosinophilia (PPI-REE). Although the underlying mechanism of PPI-REE is still unclear, it is suggested that PPIs block permeation of the causal allergens from the esophageal lumen to the subepithelium by curing the acid damage [65, 66] and that they may reduce eosinophilic inflammation by suppressing Th2-associated cytokine or gene expression independently of the gastric acid inhibitory effect [67, 68]. Thus, symptomatic and histological resolution of esophageal eosinophilia by PPIs does not necessarily indicate the existence of GERD [16]. Besides PPI therapy, topical therapy by swallowed inhaled (into the pharynx) corticosteroids (1–2 mg budesonide twice daily, a standard treatment for asthma) is applied for eosinophilic esophagitis [16]. Recently, budesonide has also been available as orodispensible tablets and suspension for the treatment of this condition [69, 70]. Studies have demonstrated that topical steroid therapy can lead to histological remission in 15–94%, and to symptomatic remission in 30–97% of patients [71]. A discrepancy between symptomatic and histological remission may be attributable to the limited efficacy of steroid therapy on fibrostenotic changes in the subepithelium [72, 73]. Dilatation therapy is recommended for symptomatic patients with esophageal strictures or narrowing despite medical therapy [16] but must be used with great caution and only by experienced physicians.

New therapeutic approaches with antibodies against IL‑5, IL‑4 or IL-13, which have been designed to target specific immune response pathways associated with eosinophilic esophagitis are currently not recommended in the guidelines of the American Gastroenterological Association Institute and the Joint Task Force on Allergy-Immunology Practice Parameters [74].

### Dr. A.J. Eherer:

After the diagnosis of eosinophilic esophagitis had been established in the discussed patient, he was treated with topical steroids and a modified elimination diet, which led to symptomatic and histological remission. However, surveillance endoscopy showed that the crepe paper-like esophagus and its increased vulnerability persisted even when he was asymptomatic on steroid therapy. Rarely, these changes may on instrumentation lead to perforation of the esophagus with the consequent need for esophagectomy [75]. Endoscopists should always be aware of this increased vulnerability when performing surveillance esophagoscopy or dilatation of esophageal strictures in patients with eosinophilic esophagitis.

### Dr. G.J. Krejs:

First described in the 1990s by Attwood [76] and Straumann [77], eosinophilic esophagitis is now an important diagnosis. Currently, about 30 adult patients are being treated in the outpatient gastroenterology clinic of the Department of Internal Medicine at Graz University Medical Center, but it is expected that this number will increase in the future.

## Final diagnosis

- Eosinophilic esophagitis
